# Interdisciplinary Nanomaterials for Biomedical Imaging and Sensing Applications

**DOI:** 10.3390/nano16010021

**Published:** 2025-12-23

**Authors:** Xinyu Chen, Ashley H. Fung, George Luka, Anthony A. Fung

**Affiliations:** 1 Department of Neuroscience, University of California San Diego, La Jolla, CA 92092, USA; xic106@health.ucsd.edu; 2 Department of Medicine, Yale School of Medicine, New Haven, CT 06520, USA; 3 Department of Electrical and Computer Engineering, University of California, Santa Cruz, CA 95064, USA; 4 Department of Biomedical Engineering, Yale University, New Haven, CT 06520, USA

**Keywords:** nanomaterial, biomedical imaging, biosensing

## Abstract

Recent advances in nanomaterials have profoundly transformed the landscape of biomedical imaging and analytical detection. By leveraging unique physical, chemical, and optical properties at the nanoscale, these materials enable unprecedented sensitivity, resolution, and specificity in both in vitro and in vivo applications. An analysis of the nanomaterials topic collection in Web of Science reveals that although biomedical imaging is not the most dominant domain in the nanomaterials field, it is rapidly growing across many disciplines and applications, including super-resolution nanoscopy, spatial sequencing technology, and immunology. Based on citation network topology and high-level topic modeling, we highlight recent advances in signal enhancement, targeted specificity, and mechanical innovations across multiple modalities, including optical imaging, contrast MRI, cytometry, and spatial sequencing.

## 1. Introduction

**Figure 1:**
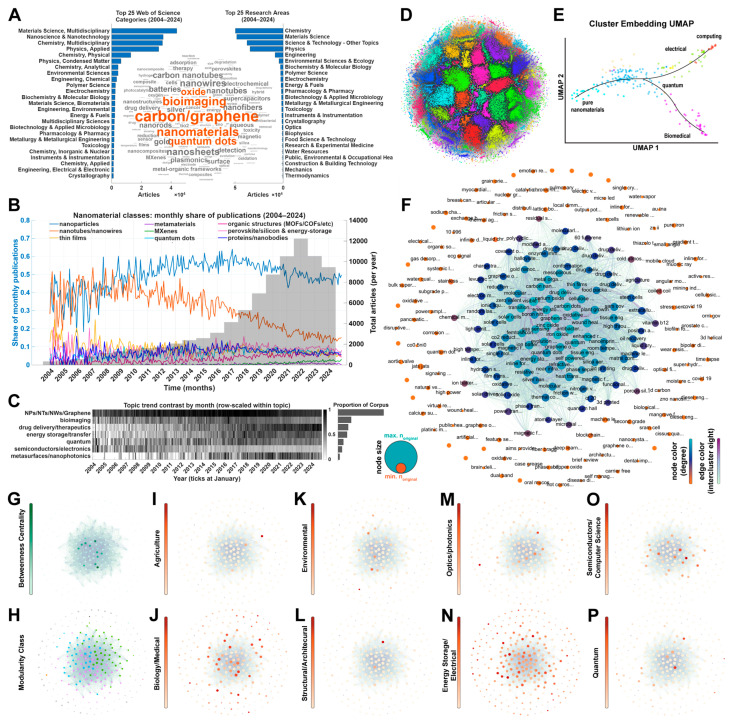
Analyzing the interdisciplinary nature and topic trends of the nanomaterials field. Materials and Methods. (**A**) Top subject matter and pooled unigrams/bigrams from abstracts of all 179,070 WoS articles (2004–2024). (**B**) Proportions of selected nanomaterial classes over time, overlaid on a histogram of total articles per year. (**C**) Topic proportion trends over time; bin intensity scales to raw counts per period divided by total articles in that period. (**D**) Citation network of Crossref articles indexed with “nanomaterial(s)” over the last five years. Edges are citation events between articles (nodes). Layout: DrL, force-directed; colored via custom k-nearest-neighbor community detection. Papers without citations or references are excluded; the network comprises 740,169 nodes and 998,098 edges. (**E**) All detected communities are projected via UMAP and recolored using a density-based scan after embedding titles, abstracts, and preprocessed full text (where available). (**F**) Communities from (**D**) pooled and re-plotted with a Fruchterman–Reingold layout (default Gephi 0.10 settings) to examine inter-group structure. Approximately half of communities exhibit only intra-community citations (degree = 0; shown in orange). (**G**) Nodes in (**F**) recolored by betweenness centrality; high-betweenness nodes frequently coincide with higher edge weights. (**H**) Nodes in (**F**) recolored by modularity class (resolution = 1). Four main classes (>1% of the network) are observed. (**I**–**P**) Nodes recolored by cosine similarity to selected subject matter, indicating mixing rather than self-aggregation across fields.

Nanomaterials are employed in many applications and have demonstrated particularly powerful utility in biomedical imaging, enabling deeper imaging, finer resolution, and enhanced contrast. Publications have surged since the early twenty-first century as synthesis and manufacturing have advanced. We analyzed the nanomaterials topic Core Collection in Web of Science (WoS) (2004–2024; 179,070 docs), summarized top categories, and tokenized abstracts (Figure 1A). Class proportions over time show dominant contributors, such as “nanoparticles”, and rising nanomaterials, such as “organics” (including metal–organic frameworks (MOFs), which were the topic of the 2025 Nobel Prize in Chemistry) (Figure 1B). Semantic screening highlights bioimaging’s steady growth and the value of context beyond category lists (Figure 1C).

To probe interdisciplinarity, we built a five-year Crossref citation network (nodes = papers; edges = citations; exclusions noted) and clustered it (Figure 1D) [1]. Communities (n = 227) were embedded and mapped with a Uniform Manifold Projection (UMAP) (Figure 1E), then re-laid out in a citation network (nodes = clusters of papers from the previous step) to inspect inter-group structure (Figure 1F). Patterns in betweenness and edge weights emerge (Figure 1G), along with four main modularity classes (resolution = 1) and high-authority groups (Figure 1H). Field similarity coloring shows mixing rather than self-aggregation (Figure 1I–P). Results indicate that these fields do not self-aggregate but rather mix, highlighting the notion that nanomaterials may have extremely wide uses and that the field as a whole may be exceedingly interdisciplinary.

The overarching aim of biomedical imaging and sensing is to observe, quantify, and interpret the molecular and cellular processes of biological function and disease. Biological systems are inherently dynamic, spatially heterogeneous, and chemically complex; therefore, effective sensing requires translating subtle biochemical or biophysical interactions into measurable and interpretable signals. Regardless of modality, the foundational principle remains the same: a biological event must be transduced into a detectable readout—whether optical, electrical, mechanical, or magnetic—with sufficient sensitivity, specificity, and temporal resolution to capture physiologically relevant information.

At the core of most sensing strategies lies the transformation of a biochemical interaction into a physicochemical change. This may involve variations in fluorescence intensity or lifetime, changes in electrical impedance, shifts in inelastic scattering and resonance, or modulation of magnetic relaxation properties. Across modalities, sensors often target a set of recurring biological cues: biomolecules (e.g., nucleic acids, proteins, metabolites), microenvironmental parameters (pH, oxygen, tension, redox state, ion concentrations), cellular behaviors (calcium signaling, membrane potential, enzyme activity), and tissue-level attributes (mechanical stiffness, vascular permeability). To detect these targets, sensing platforms employ diverse physical principles such as absorption/emission spectroscopy, inelastic scattering, photoacoustic conversion, physical deformation, and electrochemical reactions.

The design and performance of modern sensors increasingly rely on interdisciplinary integration, where materials science, chemistry, physics, engineering, and biology converge to optimize signal transduction. For instance, nanomaterials with engineered plasmonic, photonic, or catalytic properties can amplify weak biological signals far beyond the capability of bulk materials [2]. Similarly, microfluidic integration enables single-cell or subcellular research with more precise manipulation [3], while computational modeling aids in designing nanostructures with predictable optical or mechanical responses [4]. Ultimately, the performance of a sensing system emerges from the interplay of these interdisciplinary components. Enhancing sensitivity may require coupling advanced nanomaterial geometries with high-gain optical readouts, while achieving real-time, in vivo compatibility may rely on combining materials with favorable pharmacokinetics, advanced imaging hardware, and machine learning-guided signal extraction.

**Figure 2:**
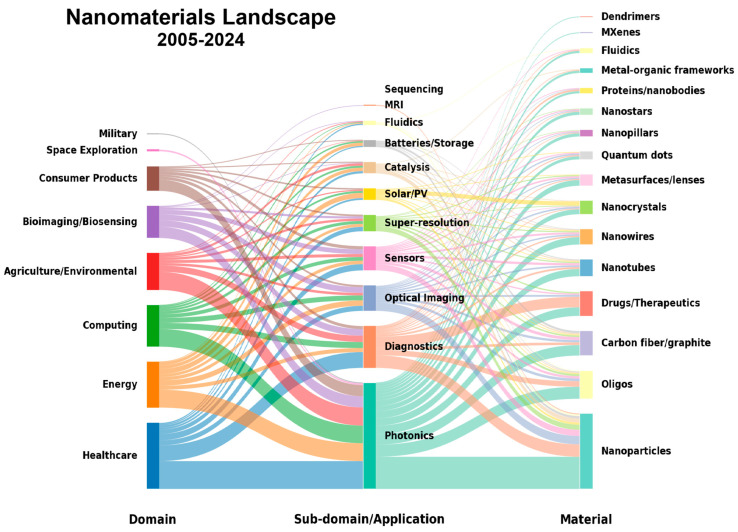
Hierarchy of nanomaterials research over two decades. Unsupervised LDA topic modeling and manual annotation of broad domains in the nanomaterials literature comprising articles with reference to several sub-domains and applications, each of which comprise a mix of different classes of nanomaterials, some of which cross-pollinate more than others.

Due to the interdisciplinary breadth of nanomaterials, this review makes a case for the growing topic share of biomedical imaging and biosensing in the field of nanomaterials by highlighting a few top nanomaterial classes (Figure 2). We discuss recent advances and their utility in improving signal-to-noise ratios, modulating the behavior of light, and improving contrast detection and specificity. Finally, we conclude with speculation on the future rise in novel spatial biology methods such as spatial mechanomics, spatial glycomics, and spatial perturbomics, and their necessity for nanomaterial development.

## 2. Materials and Methods

### Literature Corpus Analysis

All literature data analyzed in this manuscript were generated using python 3.12 in Google Colab, MATLAB R2025a, and Gephi 0.10. The WoS Core Collection under the “nanomaterials” topic was downloaded manually in batches using the CSV full export function on the website and then pooled. Crossref articles used for citation network topology analysis were retrieved using the Crossref REST API. Missing metadata such as publication year, title, abstract, and keywords were retrieved using the SemanticScholar API for all papers. Editorials, obituaries, and other similar articles were discarded, along with those that has been retracted or were otherwise missing significant metadata.

These metadata (titles, abstracts, and keywords) were used to generate nanomaterial class subject popularity and topic trend plots. The text was standardized by case, plurality, and punctuation, such as dashes or spaces. To reduce memory usage, low-topicality stop-words were removed in accordance with Zipf’s law, using a combination of unique English stop-word lists from Scikit-Learn and the Natural Language Toolkit. Articles were binarized for each nanomaterial class and topic based on whether their metadata contained relevant texts.

Citation network topology was obtained from Crossref. Each node represents one paper, and each edge represents a citation event. Each edge has a weight of 1, and it was treated as undirected, due to the fact that a paper can only cite another paper once and the cited paper has to exist before the citing paper. The network layout was generated using the Yifan-Hu algorithm, an unsupervised force-directed algorithm based solely on network topology. Communities of papers (“clusters”) were defined and colored via Louvain clustering. These clusters represent highly related papers and were subsequently plotted as individual nodes for clarity in Figure 1F using the Fruchterman–Reingold algorithm. Edge weights between these nodes is the sum of edges between communities in the Yifan-Hu network. Some communities of papers never cited other communities of papers. To model each node’s topicality, we performed term frequency–inverse document frequency (TF-IDF) calculations for each keyword and field of study (such as agriculture, electronics, energy, and bioimaging). Since these words may not be present explicitly in metadata, we averaged these scores with posterior assignment probabilities from both an error-correcting output codes (ECOCs) and long short-term memory (LSTM) classifier, using word2vec text embeddings of pooled metadata from all papers within each node (community of papers). These data were plotted as both an UMAP (Figure 1E) and used to color scale the nodes in Figure 1I–P. Node colors in the UMAP correspond to k-means clustering of the topic probabilities of each node. The number of clusters was arbitrary, but they were used to help group nodes and manually assign topic annotations.

The topical hierarchy was generated from all article metadata, with each article assigned probabilities for each domain, sub-domain, and nanomaterial class. Scores were binarized by a 50% threshold. The number of bars in each column were chosen manually based on author expertise and sampling of the literature corpus. This choice is confirmed by Latent Dirichlet Allocation (LDA) topic modeling, which uncovered an excess number of categories for each hierarchy that were manually annotated. Categories that contained duplicates were eventually decompiled into the categories shown, with little to no “other” or “no-confidence” instances.

## 3. Nanomaterials for Biomedical Imaging

### 3.1. Metasurface-Enhanced Detection and Specificity

**Figure 3:**
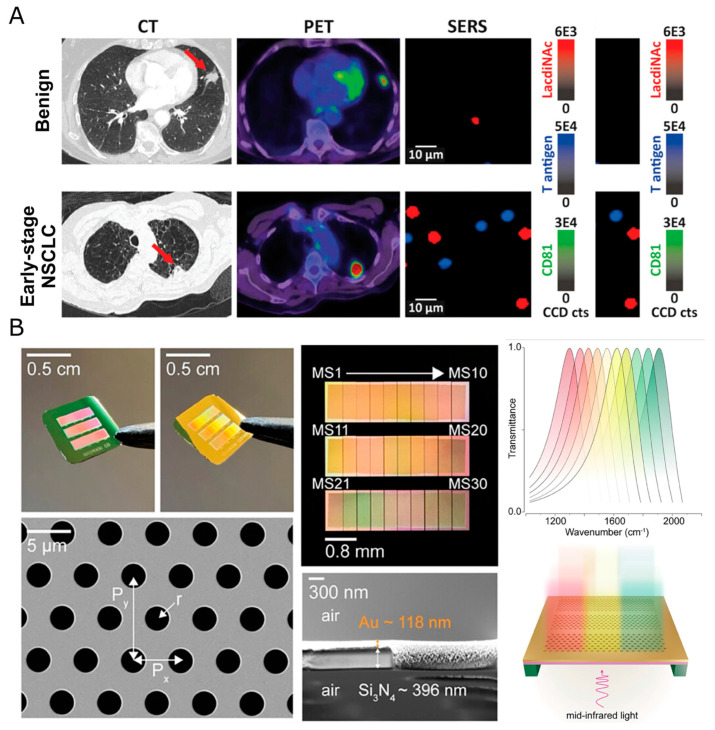
Plasmonic metasurfaces enhance spectral specificity. (**A**) sEV GLYcan PHenotype (EV-GLYPH) liquid biopsy assay detects aberrant lung cancer glycan phenotypes in human patients. Red arrows indicate nodules in patient lungs with both benign diseases and non-small cell lung carcinoma. Adapted from [16] under a Creative Commons Attribution 4.0 International License. (**B**) Gradient metasurfaces reach wafer-scale production and can enhance specific wavenumber regions spatially. Adapted from [8] under a Creative Commons Attribution 4.0 International License.

Metasurfaces are patterned arrays, often at subwavelength scales, engineered to modulate the amplitude, phase, or polarization of light. In plasmonic metasurfaces, the arrangement of “meta-atoms” within a metasurface can localize electromagnetic fields into subwavelength volumes, which can dramatically increase light–matter interactions to boost signals in chemical sensing. This is observed in both scattering spectroscopy, such as Surface-Enhanced Raman Scattering (SERS) [5,6,7], and absorption spectroscopy, such as surface-enhanced infrared absorption spectroscopy (SEIRAS) [8]. Raman nanoscopy has achieved single-molecule detection for several years and has recently seen several novel biomedical applications, including microplastic pollution, organelle phenotypic states, label-free histopathology, and glycomics [9,10,11,12]. To stress the need for nanoscale interrogation, viruses, including severe acute respiratory syndrome coronavirus 2 (SARS-CoV-2) and human immunodeficiency virus (HIV), are heavily decorated with glycoproteins, making it difficult for antibody adjuvants to bind [13,14]. Given the diversity of post-translational modifications, like glycosylation, there is significant demand for the ability to visualize them. Recently, SERS demonstrated the ability to visualize these post-translational modifications (PTMs) in vitro and ex vivo sEVs (Figure 3A) [15,16].

Despite these capabilities, the clinical translation of metasurfaces faces significant hurdles that necessitate a critical evaluation of material platforms. While plasmonic metasurfaces offer extreme field localization for sensing, they suffer from high Ohmic losses and heat generation compared to their dielectric counterparts (e.g., silicon or titanium dioxide), which can be preferable for high-efficiency imaging and lensing applications [17]. Furthermore, SERS-based sensing is often plagued by the “SERS uncertainty principle,” a trade-off where maximizing field enhancement often exacerbates signal heterogeneity due to random “hotspot” distribution [18]. This underscores the critical importance of interdisciplinary nanoparticle engineering: raw physical enhancement is insufficient without robust surface chemistry. Recent innovations have focused on “stealth” functionalization using zwitterionic polymers to prevent the non-specific fouling of proteins in complex biological fluids, a phenomenon that otherwise blinds the sensor [19,20]. Consequently, the field is moving toward hybrid systems where the metasurface dictates the optical mode, while the interdisciplinary nanoparticle interface manages biological specificity and stability [21].

Although plasmonic nanomaterials can enhance spectral quality overall, lending greater statistical power to spectral unmixing and molecular identification through the reduction in noise, nanomaterials can also be functionalized to bring specific chemical species closer to the nanomaterials that act as reporters. In these examples, the nanomaterial is either functionalized with a boronic acid substrate that serves as a binding receptor for hydroxyl groups of glycoproteins [15] or coated with anti-mucin antibodies that can be further barcoded with lectins or other antibodies to afford greater multiplexing capabilities [16]. Over the past year, advanced manufacturing has brought gradient metasurfaces to wafer-scale production. These gradient plasmonic metasurfaces have numerous advantages, including an amide 1 signal-to-noise improvement of ~20 times compared to standard CaF_2_ slides and the ability to smoothly sweep protein/lipid/carbohydrate fingerprints, instead of having a single resonance amplifier at every spot that enhances a narrow mid-IR frequency. In tests, researchers found that by spin-coating the peritoneal fluid onto the metasurface, they avoided the “coffee-ring” non-uniformity seen with drop-casting on CaF_2_ slides, yielding more consistent spectra across the field of view (Figure 3B). Metasurfaces can also leverage predetermined nanoscale patterns, such as bowties, holes, and rings, to create fixed hotspots for trapping and sensing extremely small biomolecules [22]. Plasmonic tweezers can also serve as physical modes for the precise manipulation of subcellular organelles and even other nanoparticles themselves.

Beyond signal enhancement, dielectric metasurfaces can also enable more compact lenses and on-chip processing. Miniaturizing imaging hardware without sacrificing performance is a critical consideration for intraoperative applications or wherever space is limited. High-index mid-IR metalenses have demonstrated centimeter-scale apertures for thermographic cameras with only millimeters of thickness [23]. Even in more complex microscopes that use multiple wavelengths such as Stimulated Raman Scattering (SRS), a hybrid refractive metalens demonstrated the ability to achromatically focus the pump and Stokes beams, producing higher Strehl ratios and tighter focus [24]. Metalenses can also be integrated directly onto sensors for on-chip processing. A metasurface-based full-Stokes Polarimetric Imaging sensor (MetaPolarIm) is a ~600 nm metasurface polarization filter array (MPFA) integrated with CMOS fabrication that demonstrates full-Stokes polarimetric imaging and a field of view of up to 40-degrees [25]. There are several considerations, such as optical diffraction in smaller pixel sizes, material compatibility in manufacturing with silicon sensors, and crosstalk–stability relationships with film thickness and field of view, so these demonstrations are important for future optimization. In tissue imaging, penetration depth is a major concern, especially for optical imaging. Diagnostic power increases with observable volume, and the ability to conduct 3D label-free histopathology is a major thrust in this effort and has been demonstrated using multiple technologies, such as photoacoustic imaging [26], optical coherence tomography [27], and coherent Raman scattering [11]. Typically, this is addressed through careful choice of wavelength to minimize scattering [28] or autofluorescence [29], or by well-positioned sensors that capture as much scattered light as possible [30]. However, for some applications, the polarization of light is also a critical consideration. For example, label-free imaging of collagen using Second-Harmonic Generation (SHG) can be performed in 3D due to its non-centrosymmetric structure. In that same vein, signal efficiency is heavily dependent on fiber orientation. Although the raw penetration depth of light does not increase with polarization, linearly polarized light enables the quantification of fiber angles. Circularly polarized light (CPL) averages directional sensitivity, but can be more effectively generate coherent collagen signals and has also been shown to be less susceptible to depolarization than linearly polarized light. Elliptically polarized light strikes a good balance between imaging depth and directionality [31]. Chiral metasurfaces, inspired by stomatopod eyes, have recently demonstrated full-Stokes polarization on-chip using a silicon metasurface quarter-wave plate (QWP) on top of a gold nanowire linear polarizer, separated by a SiOx dielectric spacer [32]. This advancement is a scalable and CMOS-compatible high-index metal–dielectric metasurface that offers high transmission and contrast and integrates several fabrication methods, including Plasma-Enhanced Chemical Vapor Deposition (PEVD) for the spacer layer, Reactive Ion Etching (RIE) for the Si nanopillars, and Metal Lift-Off for the Au nanowires.

### 3.2. Nanoparticles for Targeted Imaging and Tracing

**Figure 4:**
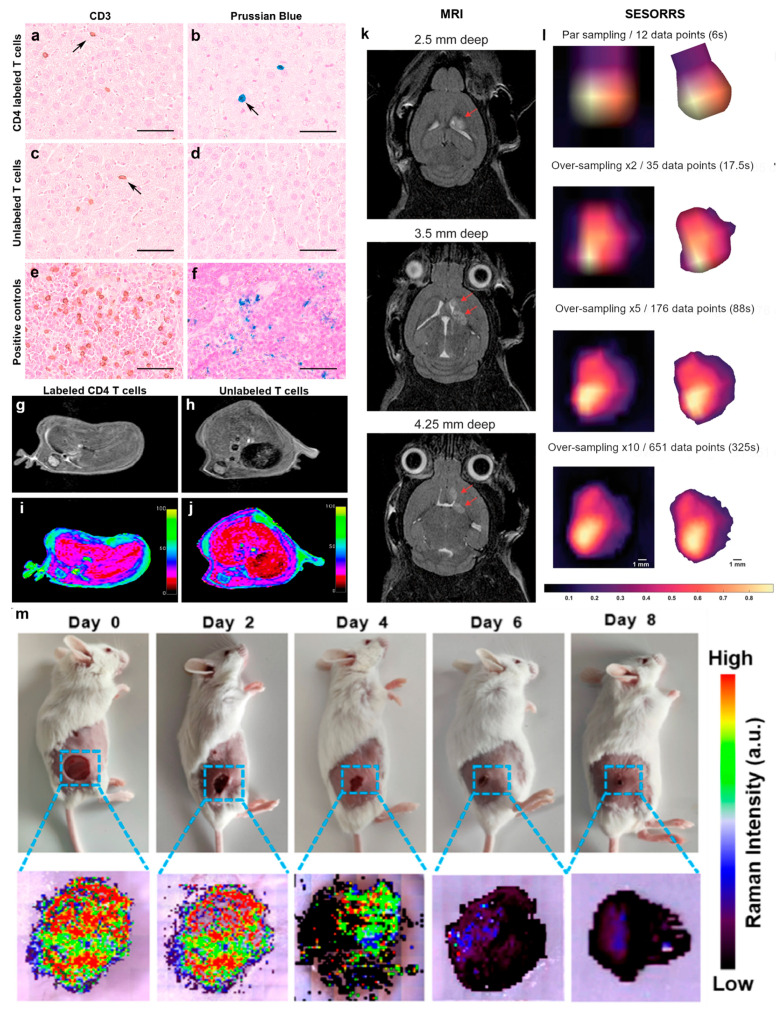
Nanomaterials enabling targeted bio-orthogonal imaging. (**a**–**f**) Histological staining of liver (**top**) and spleen (**bottom**) tissue using SPION or Prussian blue stain illustrates the distribution of T cells (arrows). (**g**–**j**) Longitudinal in vivo MR images of mice receiving intravenous injection of CD4-SPION-labeled T cells 72 h post-IV injection. (**a**–**j**) Adapted from [33] under a Creative Commons Attribution 4.0 International License. (**k**) MRI at two weeks post-injection of GL261-Luc mouse brain confirming tumors at different depths, indicated by red arrows. (**l**) SESORRS images of gold nanostar probes after background removal and radial orientation at different acquisition times. (**k**,**l**) Adapted from [34] under a Creative Commons Attribution 4.0 International License. (**m**) In vivo SERS imaging of AuNR@iQ2 NPs showing the elimination for MDRAB biofilm-infected wounds over eight days. Reproduced from [29] with permission from the relevant publisher.

In addition to signal enhancement, nanomaterials offer targeted specificity for biomedical imaging. Contemporary biomedical imaging faces a fundamental trade-off between resolution, penetration depth, and contrast sensitivity. While modalities like Magnetic Resonance Imaging (MRI) and Computed Tomography (CT) offer excellent anatomical depth, they frequently suffer from low sensitivity to molecular markers. Nanomaterials bridge this gap by acting as interdisciplinary contrast agents, where physical properties are tuned by particle size and composition, while biological specificity is dictated by surface chemistry. In optical regimes, the scattering and absorption cross-sections of spherical nanoparticles in homogeneous media are governed by Mie theory, where a slight increase in radius can dramatically enhance signal but potentially limit tissue penetration due to attenuation. Conversely, in MRI, the contrast mechanism relies on the magnetic susceptibility of the core—typically superparamagnetic iron oxide—to accelerate proton relaxation times. However, the clinical utility of these agents is not merely a function of their “brightness” but of their hydrodynamic size, which dictates their pharmacokinetics and ability to escape the reticuloendothelial system (RES). While these capabilities have been demonstrated with multiple modalities, a compelling example of this approach is demonstrated in the recent work by Yu Ping and colleagues, who employed antibody-functionalized superparamagnetic iron oxide nanoparticles (SPIONs) to label CD4^+^ T cells for Magnetic Resonance Imaging (MRI) [33]. By conjugating readily available magnetic microbeads with anti-CD4 antibodies, the study achieved receptor-mediated endocytosis of SPIONs into helper T cells, allowing for stable intracellular labeling without impairing cell viability or function. The iron oxide core of the particles provided strong T2 MRI contrast, enabling longitudinal tracking of the labeled immune cells following systemic injection in a murine model (Figure 4a–j). This example illustrates how nanomaterials can bridge molecular targeting with clinically established imaging modalities like MRI, paving the way for patient-facing applications in immunotherapy monitoring, inflammation mapping, and cell-based diagnostics.

Building on this clinical potential in the realm of optical imaging, highly specific contrast in real-time and minimally invasive formats is making transformative progress. In a recent study, Nicolson et al. bring Surface-Enhanced Resonant Raman Scattering (SERRS) and SORS together with just a 2 nM dose of gold–silica nanostars (IR-780 reporter, PEGylated) to delineate tumor margins in rapid whole-head optical screening [34]. Leveraging high-NA optics and larger-core fibers with longer working distances in a 45-degree filter geometry, the authors achieved 0.5 s integrations at photosafe power densities (~6.5 mW mm^−2^). Follow-on higher sampling frequencies even revealed a secondary, deeper lesion through the intact skull, corroborated by MRI and H&E staining (Figure 4k,l). This represents a complementary optical pathway to the CD4-SPION MRI example: here, molecularly encoded Raman fingerprints on nanostars combine with depth-sensitive SORS geometry to achieve fast, multiplex-ready tumor mapping at millimeter-scale depths, pointing toward future intraoperative guidance where wide-area scouting and focused margin confirmation can be executed in one workflow. More recently, Zhou et al. developed a nanomaterial-assisted Raman probe designed for fluorescence-free SERRS [29]. Their chemical innovation centers on iQ2, a nickel bis(dithiolene) “dark quencher”, with Au nanorod SERRS probes around it, which allows for clean, fluorescence background-free monitoring of bacterial biofilms during treatment (Figure 4m). Minati et al. also investigated photothermal stability from a different angle. They used Gold nanostars (PEG-capped) carrying adsorbed doxorubicin to serve as SERS tags. Then, under 633 nm irradiation in live cells, DOX SERS peaks diminish while fluorescence rises, reporting laser-assisted, photothermal drug release in real time [35]. With the development of therapeutic nano-carriers such as aptamer origami delivery of doxorubicin [36], a similar approach would allow for near real-time, label-free tracing of intracellular drug accumulation with both spatial precision and molecular specificity, critical for precision oncology and beyond.

However, whether employed in an optical regime or otherwise, a critical analysis of these “in vivo imageable” nanoparticles reveals that clinical translation remains disproportionately low compared to academic output. This is also partially evidenced by the overall literature topology, as the majority of paper citation clusters are found early in the translational application trajectory (Figure 1E). A key bottleneck is the biodistribution–toxicity paradox. Nanoparticles larger than the renal filtration threshold (~5.5 nm) predominantly accumulate in the liver and spleen, raising concerns about long-term organ toxicity and heavy metal retention. While “ultrasmall” nanoparticles (sub-5 nm) offer a pathway for rapid renal clearance, they often suffer from reduced magnetic moments or optical cross-sections, compromising signal utility. Furthermore, the “synthetic identity” of a nanoparticle often differs from its “biological identity”; upon entering the bloodstream, a protein corona instantly coats the surface, potentially masking targeting ligands or triggering immune clearance. Finally, the manufacturing transition from milligram-scale batch synthesis to kilogram-scale Good Manufacturing Practice (GMP) production remains a significant hurdle, as slight variations in colloidal stability can drastically alter the safety profile of the final formulation.

While these nanomaterials have emphasized bio-orthogonality, they can also be used in conjunction with imaging techniques to perturb cells and study their responses. Recently, researchers from the University of California San Diego cultured cells on engineered quartz nanopillar arrays with controlled diameter and pitch and studied them using both SRS and multiphoton fluorescence microscopy [37]. Nanopillars reduced cell spreading and circularity and altered nuclear size/circularity. Metabolically, they lowered nuclear oxidative stress and de novo protein and lipid synthesis and drove higher relative lipid saturation. This demonstrates a controlled, nanoscale mechanical cue to perturb cells in targeted ways while watching the response live. In cancer metastasis, stem cell dynamics, and cell motility, for example, nanotopography can recreate stromal architectures, challenge mechanosensitive pathways, and reveal how metabolic rewiring supports invasion [38,39,40]. These insights can help guide anti-metastatic targets, microenvironment-aware drug testing, and the design of therapeutic scaffolds that steer cell behavior toward desired outcomes.

### 3.3. Nanomaterials for Super-Resolution Imaging

Confocal microscopy has been widely used in biomedical imaging over the past several decades but relies on a diffraction-limited excitation light source, which restricts the optical resolution to hundreds of nanometers, corresponding to the wavelength of light used. Although there are post-processing steps for spatial deconvolution to enhance image quality and increase lateral resolution, this often entails fine-tuning of parameters that can greatly influence the result, and pixel intensities may not be readily quantitative [10,41]. The early 21st century witnessed a flourish of super-resolution microscopy (SRM) techniques, with many of them being commercially available today. Depending on the principles of optical design, different SRM techniques also require different protocols of sample labeling. For example, samples prepared for conventional fluorescence or confocal microscopy are transferable to be imaged under super-resolved structured illumination microscopy (SR-SIM) [42]. SIM uses the high-frequency illumination to encode the high-resolution information to a low-frequency interference pattern, and images can be computationally reconstructed by capturing multiple images with the illumination pattern shifted and rotated [43,44]. Stimulated emission depletion (STED) microscopy is a multiphoton SRM technique that is compatible with antibodies or fluorescence proteins used in conventional microscopy, only requiring users to optimize the protocol of fixation, permeabilization, blocking, washing, etc. [45]. The STED beam uses a phase plate, a type of spatial light modulator (SLM) nanomaterial, to change the beam shape to deplete the fluorescent signal around the center and shrink the effective fluorescent spot size below the diffraction limit. Thus, no complicated image reconstruction is needed, as in SIM [46]. However, the high-power STED beam also brings the photobleaching problem, which means a more stable fluorescent probe is needed for long-term, dynamic observation [47]. PhotoActivated Localization Microscopy (PALM) and Stochastic Optical Reconstruction Microscopy (STORM) share the same principle of stochastic excitation of sparse points, followed by subsequent photobleaching [48,49]. Both PALM and STORM can be implemented on conventional fluorescence microscopes, but they require either photoactivatable fluorescent proteins or photoswitchable organic dyes [50]. These fluorophores are inherently nanomaterials but require a weak activation laser or specific chemical buffers to ensure sparsity and switching between “on” and “off” states.

**Figure 5:**
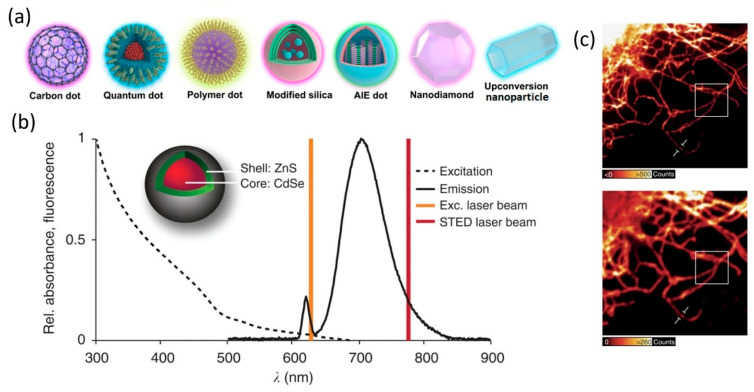
(**a**) Examples of nanomaterials for super-resolution imaging. (**b**) ZnS-coated CdSe is suitable for STED imaging. (**c**) **Top**: Vimentin fibers immunostained with QDs and imaged by STED. **Bottom**: Same region under confocal microscope. (**a**) Adapted from [51] under a Creative Commons Attribution 4.0 International License. (**b**,**c**) Adapted from [53] under a Creative Commons Attribution 4.0 International License.

To address this, small luminescent nanoparticles like carbon dots (CDs) or quantum dots (QDs) are great substitutes for traditional fluorescent dyes in SRM due to their stability and high luminescent quantum yield [51,52]. They naturally blink, making them very ideal for PALM and STORM. Recently, the successful application of QDs in STED has been demonstrated by Hell et al. [53]. They found that commercially available ZnS-coated CdSe or CdTe QDs can be excited at 628 nm with a pulse train of 38 MHz and depleted using a 775 nm pulsed laser with a 1.2 ns pulse duration, with suppressed blinking. Furthermore, they could be scanned more than 1000 times at a resolution down to ~50 nm without significant fading (Figure 5). Shortly after, another group showed that switching the 775 nm pulsed depletion laser to a 775 nm continuous-wave laser would solve the problem of re-excitation [54]. Most of the commonly seen QDs today exhibit intermittency in their emission, called the “blinking effect”, due to intrinsic properties [55]. The blinking effect can induce artifacts in STED, but, on the other hand, can become an advantage in stochastic reconstruction microscopy. As a result, super-resolution optical fluctuation imaging (SOFI), a new SRM technique, was first demonstrated using QDs [56]. SOFI requires only a conventional wide-field microscope and a CCD camera but can achieve a lateral resolution of ~50 nm and an axial resolution of ~80 nm, as reported by others [57]. Similar to QDs, CDs also exhibit fluorescence intermittency, and their application in SOFI has also been demonstrated [58]. He et al. investigated the fluorescence properties of QDs, CDs, and organic dyes like Cy3 [59]. While dye molecules are photobleached after 600 s of illumination, CDs and QDs fluorescence remain stable even after 30 min of exposure. QDs have a long “on” period and a short “off” period, whereas CDs show the opposite behavior. The low duty cycle (~0.003) of CDs thus meet the requirements for STORM imaging [59]. On the other hand, the blinking effect can be eliminated through simple surface passivation [60]. Non-blinking, non-toxic CDs showed strong performance in STED imaging in both fixed and live cells, with a resolution down to 30 nm [61]. A more detailed review of nanomaterials for SRM can be found in [51].

Tip-Enhanced Raman Scattering (TERS) is technique that leverages a gold- or silver-coated Atomic Force Microscopy (AFM) tip or solid gold or silver nanowires, along with a laser, to achieve lateral resolutions on the order of nanometers or below [62]. It is more common to use tips with incident curvatures that produce spatial maps with a resolution of 10–20 nm; however, this method may be less suited for studying biology at clinical pathology scales; simultaneous AFM and TERS can reveal biomolecular responses to physical stimuli. As many researchers race to design and model virtual cells, there will likely be a case for, and push toward, mechanotransduction and spatial mechanomics platforms.

### 3.4. Nanobodies for Multiplex Imaging

The discussed advancements in the field of super-resolution imaging offer a promising window of opportunity for biological imaging. With improved resolutions, biological systems can be studied at a finer scale, allowing for greater insight into spatial interactions and architecture within cells and tissues. Currently, immunostaining is a dominant approach for visualizing cellular components at the micro- and nanoscale. This is for good reason, as antibody labeling offers high target specificity, and their production has matured to industrial scales, leveraging natural immune surveillance processes rather than intelligent material design. However, this approach is not without limitations. The average antibody spans about 10–15 nm or 150 kDa in size, making them larger than many of the intended target antigens and introducing a sizeable separation between the targeted antigen and the visible signal from the attached marker, known as linkage error [63]. This makes accurate visualization of spatial organization at nanometer scales difficult. For example, in many applications, such as studies of histone modifications in the nucleosome, steric hindrance becomes a major concern. Oftentimes, histone marks (modifications such as methylations, acetylations, and phosphorylations on residues of histone proteins) may only be a few amino acids apart [64]. Due to the size of antibodies—although already at the nanoscale—this restriction of space prohibits the simultaneous labeling of nearby histone marks. Recently, however, there have been a number of advancements that make nanoscale imaging with antibodies more feasible. These developments span synthesis and control of antibody behavior, including their assembly and disassembly. Here, we investigate the utility of these approaches in biomedical imaging at the nanoscale.

One approach is the use of nanobodies. Nanobodies, which consist of only the heavy-chain variable region of camelid antibodies, are only about 4 nm or 15 kDa in size. This reduction in size decreases linkage error and allows for more accurate localization of biological signals. Fluorescently conjugated nanobodies have been used in place of primary antibodies with STORM imaging, allowing high-resolution of traditionally difficult-to-image structures like microtubule bundles and individual nuclear pore complex proteins [65,66]. Methods have also been developed to allow conjugation of nanobodies to oligonucleotide sequences, which allows the use of nanobodies in a more diverse array of protocols, such as DNA-PAINT [67,68].

Another frontier is the development of antibodies that can be selectively removed or deactivated after a round of imaging. While such methodologies have garnered significant attention for their use in multiplexing strategies, a less explored potential lies in their use for visualizing closely localized proteins. Recently, digestible antibodies such as REAlease probes from Miltenyi have shown promise in this arena. REAlease probes consist of antibody fragments multimerized with dextran linkers and conjugated to fluorescent labels. In the presence of dextranase, the linkers can be digested, effectively releasing the antibodies from epitopes [69]. By cyclically hybridizing and digesting these antibodies, researchers would be able to probe closely localized targets, such as histone marks, without concerns for competition (or steric hindrance).

### 3.5. Computational Algorithms for Image Resolution Enhancement

Although hardware-based super-resolution microscopy techniques have successfully surpassed the diffraction limit, these methods often involve significant costs due to intricate experimental setups. In parallel with these hardware innovations, a “software revolution” has emerged in the form of computational resolution enhancement. This field is dedicated to developing algorithms that post-process images acquired from standard, diffraction-limited microscopes to computationally enhance resolution, deblur optical artifacts, denoise images, and reduce artifacts. This approach offers a powerful, accessible, and often less invasive alternative to specialized hardware.

Early image quality enhancement methods rely heavily on math theories. Some examples are total variation regularization [70] and sparse deconvolution [71]. The former is based on the assumption that images are often composed of piecewise-smooth regions separated by sharp edges, whereas the latter is based on the fact that natural and biological images are often “sparse,” meaning they can be represented by a small number of non-zero coefficients in a suitable transform domain (e.g., wavelets); a resolution of 60 nm was reported in [71]. Machine learning techniques such as genetic algorithms and gradient descent have further enabled the deconvolution of single-frame images using pointillism assumptions. Tools such as SUPPOSe [72] and A-PoD [10] constrain the number of virtual emitters of given intensity and solve for their spatial superposition through the point spread function (PSF) of the microscope. This allows for an easy improvement in image quality and, in most cases, spatial resolution without excessive processing time or image frames. This can be necessary when imaging times are prohibitively long, because researchers can instead capture faster images while achieving similar quality to longer acquisition times and frame averaging.

The mid-2010s marked a profound paradigm shift with the advent of deep learning, particularly Convolutional Neural Networks (CNNs). Instead of explicitly modeling the physics of the PSF, deep learning models learn complex, non-linear transformations from low-quality to high-quality images directly from vast amounts of training data. From supervised CNN to generative adversarial networks, and to self-supervised or unsupervised learning, increasingly data-efficient models have been developed. For example, Noise2Void, a self-supervised denoising model, can learn to predict a pixel’s value from its noisy neighbors without the need for clean ground-truth data, dramatically expanding the applicability of deep learning in experimental settings [73]. By late 2025, the field has moved beyond standard CNNs to more powerful and sophisticated architectures capable of capturing long-range spatial dependencies, modeling complex data distributions, and generalizing across different experimental conditions. For example, SwinIR employs a hierarchical structure with shifted window-based self-attention, allowing efficient modeling of both local and global relationships within an image [74]. Its ability to capture long-range dependencies is crucial for accurately reconstructing repetitive biological structures like cytoskeletal filaments [75]. A 2024 study introduced a Physics-Informed Denoising Diffusion Probabilistic Model (PI-DDPM) for microscopy image reconstruction [76]. This model incorporates the physical forward model of light propagation (i.e., blurring and noise process) into the loss function of the deep learning model, significantly reducing artifacts and hallucinations compared to standard U-Net and diffusion models. In the future, the practical impact of these advanced algorithms should be magnified by the development of user-friendly software platforms that make them accessible to biologists and researchers without expertise in computer science.

## 4. Nanomaterials for Biosensing

### 4.1. Nanoparticles for Spatial Sequencing and Proteomics

While detecting the spatial information of one or a few proteins within biological tissues can now be easily performed, challenges still remain when it comes to large-scale profiling of proteins and RNAs due to the overlapping emission spectrum of fluorescent dyes. Over the past decade, spatial transcriptomics and proteomics have rapidly advanced, establishing themselves as powerful tools for exploring molecular organization in tissues. Many of these approaches are imaging-based, building upon decades of innovation in fluorescence in situ hybridization (FISH), first introduced in the 1980s. Meanwhile, the rise in single-cell RNA sequencing (scRNA-seq) has enabled strategies that embed spatial information directly into sequencing data, offering complementary ways to map cellular landscapes.

Early FISH probes were either indirectly labeled using a secondary antibody or directly conjugated with fluorophores [77,78]. The sensitivity of these probes was very limited given the low number of fluorescent molecules and high tissue autofluorescence. Subsequently, long RNA probes were cut into small oligonucleotides of 50–60 bases, labeled with four or five fluorophores, achieving visualization of single RNA transcripts [79]. There are many variants of single-molecule (sm)FISH, but multiplexing remained an issue until the development of seqFISH [80] and multiplexed error-robust FISH (MERFISH) [81]. Instead of using different fluorophores to distinguish between RNA transcripts, both encode transcriptome information using unique barcodes. In each imaging round of seqFISH, each RNA transcript is tagged by a single type of fluorophore, and the FISH probes are removed at the end of each imaging round. Each specific transcript will have a unique combination of fluorophore sequence (e.g., Red, Green, Blue, Green). MERFISH follows a similar principle, except that each RNA transcript is assigned a unique binary barcode (e.g., 1001110), and each barcode is separated by a minimum Hamming distance of four to make the method error-robust. Both methods can theoretically profile the whole human genome within a reasonable number of hybridization and imaging rounds.

scRNA-seq has revolutionized transcriptomic research by enabling the profiling of gene expression at the resolution of individual cells. The workflow is now well-streamlined by commercial products like 10x Genomics (Pleasanton, CA, USA) Chromium, which provides droplet-based microfluidic systems that encapsulate single cells with barcoded beads, and high-throughput sequencing platforms like Illumina NovaSeq. Over the past decade, numerous breakthroughs have enabled the integration of spatial information into single-cell transcriptomic data, many of which have leveraged the unique properties of nanomaterials. 10x Genomics Visium is one of the first commercialized spatial transcriptomic platforms [82]. It provides microarray slides coated with barcoded oligonucleotides, which are composed of spatial barcodes, UMIs, and poly-dT sequences. Tissue is permeabilized on the slide, and released mRNAs hybridize to the barcoded oligos at nearby spots. Then, cDNA molecules incorporating both transcript sequences and spatial barcodes are then generated by reverse transcription. After sequencing and reconstruction, a map of gene expression overlaid on tissue morphology can be generated. Each capture spot on the Visium slide is 55 μm. To achieve better resolution, Rodriques et al. developed Slide-seq, where DNA-barcoded 10 μm beads were deposited onto a glass slide [83]. In Slide-seqV2, they greatly increased capture efficiency by optimizing their protocols in bead synthesis, indexing, etc. [84]. Vickovic et al. further brought the resolution down to 2 μm by depositing barcoded beads into hexagonal array of 2 μm wells on silicon wafers [85].

Due to the complexity of tissue composition and cell–cell interactions, researchers are now integrating multi-omic approaches, including transcriptomics, proteomics, epigenomics, and metabolomics, to better understand biological processes like neurodegeneration, tumor microenvironment, and metabolome [86]. For example, spatial-ATAC-RNA-seq or spatial-CUT&Tag-RNA-seq could achieve genome-wide co-profiling of transcriptome and epigenome by leveraging microfluidic devices with orthogonal channels delivering two sets of oligonucleotide barcodes [87]. These techniques excel at interrogating the entire transcriptome and genome without requiring the design of pre-defined gene panels. Due to the tremendously huge amount of single-cell data, developing computational methods for multi-omics data integration is also becoming more and more crucial, and a detailed review of integration principles can be found in [88].

### 4.2. Nanomaterials for Cytometry and Lateral Flow Assays

Nanomaterials have reshaped the landscape of diagnostic technology, especially major biosensing platforms like Lateral Flow Assays (LFAs) and liquid biopsy. LFAs are paper-based, point-of-care diagnostic devices valued for their affordability, portability, and rapid visual readout. Classic examples, such as pregnancy tests and COVID-19 antigen kits, typically use spherical gold nanoparticles (AuNPs) that generate color signals through localized surface plasmon resonance (LSPR) [89]. While these assays are robust and simple to interpret, they are largely qualitative, providing only yes/no results. Moreover, their sensitivity is limited, with detection thresholds usually in the high picogram- to nanogram-per-milliliter range, insufficient for identifying biomarkers that occur at trace concentrations during early stages of disease [90]. Nanotechnology provides a pathway to overcome this barrier. Engineered nanomaterials can enhance signal generation, concentrate target molecules, and enable multiplexed or quantitative analysis, thereby bridging the sensitivity gap in traditional assays. Over the past decade, researchers have designed nanostructures with tailored optical, magnetic, and catalytic properties to expand the analytical capabilities of LFAs. Through these advances, detection limits have dropped from the high pg or ng per mL range to orders-of-magnitude lower concentrations, expanding the clinical relevance of LFAs for early disease detection.

**Figure 6:**
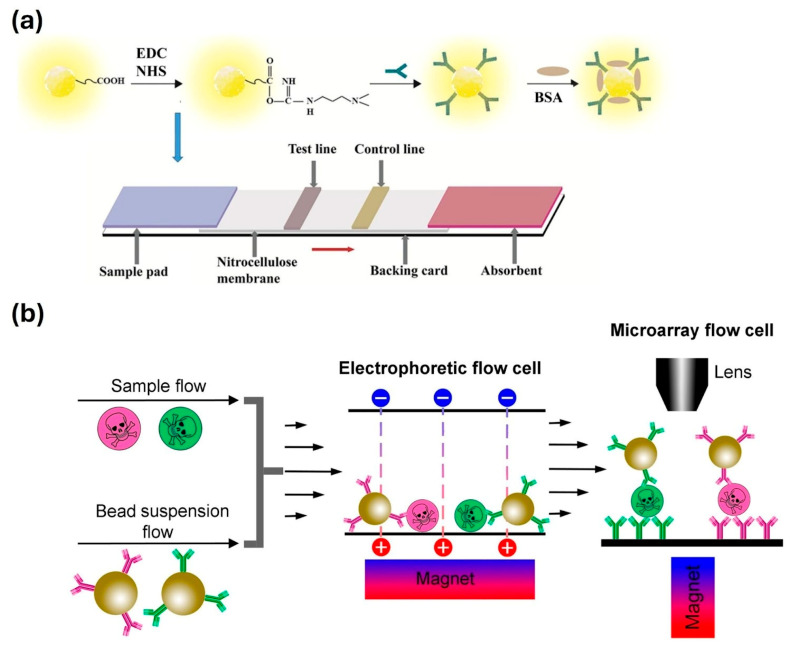
(**a**) Design of QD-based LFA. Adapted from [91] under a Creative Commons Attribution 4.0 International License. (**b**) Design of magnetic beads-based LFA. Adapted from [93] under a Creative Commons Attribution 4.0 International License.

A major strategy for improving the analytical performance of Lateral Flow Assays (LFAs) is the use of optical and fluorescent nanomaterial reporters like QDs or upconversion nanoparticles (UCNPs) in place of, or in combination with, traditional AuNPs. QD-based LFAs have achieved detection limits as low as 0.3 ng/mL for protein biomarkers such as human IgG, representing a 10–100-fold improvement over conventional colloidal gold assays [91] (Figure 6a). Similarly, UCNPs, which emit visible light upon infrared excitation, effectively eliminate autofluorescence interference and support ultra-low background detection. UCNP-based LFAs have reached sub-nanogram per milliliter sensitivity for clinically relevant biomarkers [92].

Another effective strategy for improving LFAs performance is the use of magnetic nanoparticles to actively concentrate target analytes at the test line. Superparamagnetic beads, such as Fe_3_O_4_ or FeCo nanospheres, are typically functionalized with antibodies specific to the target molecule and mixed directly with the sample. As the sample migrates along the strip, an external magnet placed beneath the test zone attracts the magnetic complexes, drawing them out of solution and onto the capture area. This magnetic focusing effect greatly increases the local analyte concentration, enhancing signal intensity and improving test reproducibility [93] (Figure 6b). Experimental studies have shown that preconcentration with magnetic nanobeads can enhance assay sensitivity by one or more orders-of-magnitude, particularly in complex biological matrices such as serum or whole blood, where passive diffusion limits molecular transport [94].

Beyond conventional metallic and fluorescent probes, a growing range of emerging nanomaterials is being incorporated into LFAs to achieve multimodal signal readouts and enhanced biorecognition efficiency. These materials combine structural versatility with unique physicochemical properties, allowing them to perform multiple functions within a single assay. Metal–organic frameworks (MOFs) and porous silica nanoparticles exemplify this trend. Their exceptionally high surface area enables dense loading of both capture ligands and signaling molecules, increasing the effective number of interactions per target and amplifying the readout signal. MOF-based LFA labels have been designed to host enzyme-mimetic catalytic sites or encapsulate large numbers of fluorophores within their porous networks, achieving marked improvements in detection sensitivity [95,96,97,98].

Equally promising are two-dimensional (2D) nanomaterials such as graphene oxide, molybdenum disulfide (MoS_2_), and MXenes (e.g., Ti_3_C_2_). These nanosheets offer tunable surface chemistry and multifunctionality, making them ideal for biosensing [99,100,101]. A recent study developed a novel multifunctional Fe_3_O_4_@MoS_2_@Pt composite nanotag featuring remarkable peroxidase-like activity, photothermal properties, and magnetic separation capability. The authors demonstrated limits of detection of 1 ng/mL for SARS-CoV-2 and 1 μg/mL for H1N1 [102].

Other emerging nanomaterials, including perovskite nanocrystals (offering bright, tunable emission), carbon dots (biocompatible fluorescent probes), and hybrid polymer nanoparticle composites, are also being explored for dual colorimetric or electrical readouts [103,104,105]. Together, these materials are expanding LFAs beyond traditional single-analyte color tests, enabling integrated, multi-signal diagnostic devices that provide rapid, accurate, and quantitative information at the point of care.

### 4.3. Nanomaterials for Other Industrial Sensing Products

The integration of nanomaterials into diagnostic and monitoring devices represents a pivotal advancement in biotechnology because nanomaterials drastically alter the physicochemical properties of sensor interfaces. These alterations are the primary drivers for profound improvements in three critical performance areas: sensitivity and selectivity, miniaturization and portability, and overall cost-effectiveness. A wide range of nanomaterials has been developed to serve as highly tunable platforms for biomedical imaging and sensing. Semiconductor quantum dots offer size-dependent emission, high brightness, and exceptional photostability, making them valuable for multiplexed fluorescence imaging and long-term tracking [106]. Carbon dots provide similar optical tunability with superior biocompatibility and low toxicity, enabling their use in intracellular sensing, pH monitoring, and reactive oxygen species detection [107]. Metal and plasmonic nanoparticles, including gold and silver nanostructures, exhibit strong localized surface plasmon resonance effects, forming the basis of ultrasensitive SERS probes and plasmonic refractive-index sensors [108]. Magnetic nanoparticles, particularly iron oxide formulations, enable contrast enhancement in MRI and can be engineered for magneto-thermal or magneto-mechanical sensing [109]. More recently, metal–organic frameworks (MOFs) have emerged as versatile hybrid structures with large surface area, tunable porosity, and modular chemical functionality that support high loading capacity—properties that make them attractive for gas storage, capture of carbon dioxide, and catalysis [110].

Materials such as AuNPs, porous carbon structures, and MOFs provide a vastly increased electroactive surface area compared with a flat, bulk material electrode of the same geometric size. This allows for a much higher density of immobilized biorecognition elements—such as enzymes, antibodies, or synthetic aptamers—per unit area. The result is a significant amplification of the electrochemical signal (e.g., current or voltage change) for a given analyte concentration, which directly translates to a lower limit of detection (LOD) and a wider linear sensing range [111]. For example, traditional sweat sensors operate on a colorimetric principle: as sweat flows through micro-channels in the patch, it dissolves non-toxic food dyes, causing color to fill the channels [112]. Researchers have found that carbon nanomaterials can enhance the performance of sweat sensing in various ways, such as increasing the electrochemical signal by providing more active sites and improving optical signal intensity by enhanced light absorption and emission [113].

The use of nanomaterials also enables advanced microscale electrode fabrication. For example, the dominant commercial glucometer technologies, as of late 2025, are based on the “wired enzyme” approach, which utilizes an osmium-based mediator polymer that is co-immobilized with the GOx enzyme. Modern continuous glucose monitors, such as the Dexcom (San Diego, CA, USA) G7, capitalize on these fine sensor filaments, in this case, a platinum and tantalum core wire, which are then coated with various functional layers [114]. On the other hand, the research frontier of glucometers is almost entirely driven by nanomaterials, with a strong focus on developing non-enzymatic sensors. This approach seeks to replace the fragile GOx enzyme with robust inorganic catalysts. Recent patents and studies describe sensors built on Laser-Induced Graphene (LIG) electrodes coated with bimetallic layers of nickel and gold [115]. These materials directly catalyze glucose oxidation, offering the potential for indefinite shelf life and resilience to temperature fluctuations—critical advantages for improving access in regions with challenging supply chains. Meanwhile, MXenes, a rapidly growing family of 2D transition metal carbides and nitrides, offer exceptional electrical conductivity, hydrophilicity, and surface chemistry versatility, supporting their use in energy storage and harvesting, water purification, and wearable/implantable biosensors [116]. A 2025 study on a flexible graphene fiber glucose sensor decorated with gold and nickel hydroxide demonstrated a remarkable detection limit of 0.294 µM and robust stability over 14 days of continuous testing [117]. Together, these nanomaterial platforms exemplify the field’s ability to tailor optical, electronic, and catalytic properties at the nanoscale to achieve enhanced contrast, sensitivity, and functional specificity in complex biological environments. These small sensors are key to the development of minimally invasive devices like microneedle arrays that sample interstitial fluid without causing pain or significant tissue damage [118] and may be a critical consideration for the potential future conjugation of multiple nanomaterial devices that enable both sampling and analysis.

While the synthesis of certain advanced nanomaterials can be expensive at the laboratory scale, their application in the manufacturing of diagnostic devices often leads to a significant reduction in overall unit costs and capital expenditure. A primary driver of cost reduction is the shift in manufacturing paradigms. Traditional fabrication of high-performance electrodes often relies on photolithography, a multi-step process that requires expensive equipment, high-vacuum environments, and sterile cleanroom facilities. In contrast, nanomaterial-based conductive inks are compatible with high-throughput, atmospheric pressure techniques such as screen printing, inkjet printing, and roll-to-roll processing [119]. In addition, nanomaterial-modified electrodes can simplify the supporting electronics. The stronger signal-to-noise ratio often reduces the need for complex, high-gain amplification circuits in the reader device, allowing the use of cheaper, lower-power electronic components [2].

## 5. Conclusions

Nanomaterials are redefining biomedical imaging by coupling physical amplification (field confinement, resonance engineering, and photostability) with biological selectivity (targeted ligands, bio-orthogonal chemistries, and nucleic acid barcodes). The advances surveyed here highlight how material design at the nanoscale is now inseparable from method design in modern spatial biology. Together, these platforms increase sensitivity (via enhanced fields and catalytic signal gain), specificity (via precise bioconjugation and barcoding), and throughput (via multiplex detection and integrated, chip-scale optics). Additionally, while their namesake implies the ability to enter small places, steric hindrance may still prevent high-resolution multiplexing when protein probes are necessary. Recent developments have addressed this limitation by engineering nanobodies and commercial digestible antibodies. Whether biologically inspired or purely inorganic, nanomaterials have increased both the breadth of applications and targeted specificity of biomedical imaging.

Moreover, in the biomedical field, there is a momentous push toward spatial context over bulk methods. In response, new technologies are on the rise, including spatial glycomics, spatial mechanomics, and spatial perturbomics. Without a doubt, careful consideration of nanomaterials will be tandemly present at every step. For instance, the ability to resolve glycoproteins in situ, as discussed above, has seen marked improvement thanks to resonance enhancement of spectroscopic techniques mentioned above; however, label-free methods such as matrix-assisted laser desorption ionization (MALDI) mass spectrometry still dominate despite lower spatial resolution. This is because of the huge variety of glycans and other post-translational modifications, some of which, such as lectins, are present on commercial antibody probes. This precludes multiplexing capabilities and underscores the need for further progress in simultaneous protein and glycan staining for high-resolution optical imaging. In spatial mechanomics, a nano-tip, often made from diamond, is used to deform the tissue specimen in a process called nanoindentation. In a setup similar to Atomic Force Microscopy (AFM), this process is regularly patterned across the analyte tissue to derive its biomechanical properties [120]. Finally, in the age of artificial intelligence, many researchers and consortia are racing to build models of virtual cells, which require careful metabolic rate kinetics data and complex non-linear simulations of responses to perturbations. Clustered Regularly Interspaced Short Palindromic Repeats (CRISPRs) is an excellent method for high-throughput perturbation screening, but this typically takes the form of bulk methods resolved stochastically rather than in situ. While commercial platforms like Xenium (10x Genomics) allow for the visualization of gRNAs, this requires the pre-determination of a gRNA panel of interest, which can be increasingly expensive with larger gRNA panels. Therefore, there is a strong need for methods that enable deep sequencing of perturbations with spatial context at low cost. These are prime examples of interdisciplinary nanomaterials involving multiple modalities and are some of the most frequently discussed classes of nanomaterials observed in the recent literature. Nevertheless, other nanomaterials such as lipid nanoparticles can be useful in metabolic imaging as well, such as tracking de novo lipogenesis using isotopic metabolites [121,122,123,124], potentially allowing for their bio-orthogonal tracking of drug delivery without other probes that might influence distribution. Although the literature on these methods is currently sparse, their coalescence of spatial biology and nanomaterials is undeniable.

## Data Availability

The original data presented in the study are openly available at https://doi.org/10.5281/zenodo.17593546.

## Abbreviations

The following abbreviations are used in this manuscript:

AFM	Atomic Force Microscopy
Ag	Silver
Au	Gold
AuNPs	Gold Nanoparticles
CDs	Carbon Dots
CNNs	Convolutional Neural Networks
CPL	Circularly Polarized Light
CRISPR	Clustered Regularly Interspaced Short Palindromic Repeats
CT	Computed Tomography
DOX	Doxorubicin
ECOC	Error-Correcting Output Codes
EV-GLYPH	sEV GLYcan PHenotype
FISH	Fluorescence In Situ Hybridization
GMP	Good Manufacturing Practice
HIV	Human Immunodeficiency Virus
iQ2	Tetrabutylammonium bis(bis(4-methoxyphenyl)dithiocarbamato)nickelate
LDA	Latent Dirichlet Allocation
LFAs	Lateral Flow Assays
LIG	Laser-Induced Graphene
LOD	Limit of Detection
LSPR	Localized Surface Plasmon Resonance
LSTM	Long Short-Term Memory
MALDI	Matrix-Assisted Laser Desorption Ionization
MERFISH	Multiplexed Error-Robust FISH
MetaPolarIm	Metasurface-Based Full-Stokes Polarimetric Imaging Sensor
MOFs	Metal–Organic Frameworks
MoS2	Molybdenum Disulfide
MPFA	Metasurface Polarization Filter Array
MRI	Magnetic Resonance Imaging
OCT	Optical Coherence Tomography
PALM	Photoactivated Localization Microscopy
PEVD	Plasma-Enhanced Chemical Vapor Deposition
PI-DDPM	Physics-Informed Denoising Diffusion Probabilistic Model
PSF	Point Spread Function
PTMs	Post-Translational Modifications
QDs	Quantum Dots
QWP	Quarter-Wave Plate
RES	Reticuloendothelial System
RIE	Reactive Ion Etching
SARS-CoV-2	Severe Acute Respiratory Syndrome Coronavirus 2
scRNA-seq	Single-Cell RNA Sequencing
SEIRAS	Surface-Enhanced Infrared Absorption Spectroscopy
SERRS	Surface-Enhanced Resonant Raman Scattering
SERS	Surface-Enhanced Raman Scattering
SESORS	In Vivo Imaging Using Surface-Enhanced Spatially Offset Raman Spectroscopy
SHG	Second-Harmonic Generation
SIM	Structured Illumination Microscopy
smFISH	Single-Molecule FISH
SOFI	Super-Resolution Optical Fluctuation Imaging
SORS	Spatially Offset Raman Spectroscopy
SPIONs	Superparamagnetic Iron Oxide Nanoparticles
SR-SIM	Super-Resolved Structured Illumination Microscopy
SRM	Super-Resolution Microscopy
SRS	Stimulated Raman Scattering
STED	Stimulated Emission Depletion
STORM	Stochastic Optical Reconstruction Microscopy
TERS	Tip-Enhanced Raman Scattering
TF-IDF	Term Frequency–Inverse Document Frequency
UCNPs	Upconversion Nanoparticles
UMAP	Uniform Manifold Projection
WoS	Web of Science
